# Allocating Scarce Resources Strategically - An Evaluation and Discussion of the Global Fund's Pattern of Disbursements

**DOI:** 10.1371/journal.pone.0034749

**Published:** 2012-05-09

**Authors:** David McCoy, Kelvin Kinyua

**Affiliations:** 1 Aidspan, Nairobi, Kenya; 2 Centre for International Health and Development, University College London, London, United Kingdom; University of California, San Francisco, United States of America

## Abstract

**Background:**

The Global Fund is under pressure to improve its rationing of financial support. This study describes the GF's pattern of disbursements in relation to total health expenditure (THE), government health expenditure (GHE), income status and the burden of HIV/AIDS, TB and malaria. It also examines the potential for recipient countries to increase domestic public financing for health.

**Methods:**

This is a cross-sectional study of 104 countries that received Global Fund disbursements in 2009. It analyses data on Global Fund disbursements; health financing indicators; government revenue and expenditure; and burden of disease.

**Findings:**

Global Fund disbursements made up 0.37% of THE across all 104 countries; but with considerable country variation ranging from 0.002% to 53.4%. Global Fund disbursements to government amounted to 0.47% of GHE across the 104 countries, but again with considerable variation (in three countries more than half of GHE was based on Global Fund support). Although the Global Fund provides progressively more funding for lower income countries on average, there is much variation at the country such that here was no correlation between per capita GF disbursements and per capita THE, nor between per capita GF disbursement to government and per capita GHE. There was only a slight positive correlation between per capita GF disbursement and burden of disease. Several countries with a high degree of 'financial dependency' upon the Fund have the potential to increase levels of domestic financing for health.

**Discussion:**

The Global Fund can improve its targeting of resources so that it better matches the pattern of global need. To do this it needs to: a) reduce the extent to which funds are allocated on a demand-driven basis; and b) align its funding model to broader health systems financing and patterns of health expenditure beyond the three diseases.

## Introduction

The Global Fund (GF) is one of the major sources of external development assistance for health (DAH) worldwide. As of the end of 2010, it had committed US$ 21.7 billion in 150 countries to support large-scale prevention, treatment and care programs against three diseases [1]. Its proportional contribution to DAH has risen from 1% in 2002 to 11% in 2010 [2] at which point its annual disbursements reached US$ 3 billion for the first time [1].

**Figure 1:**
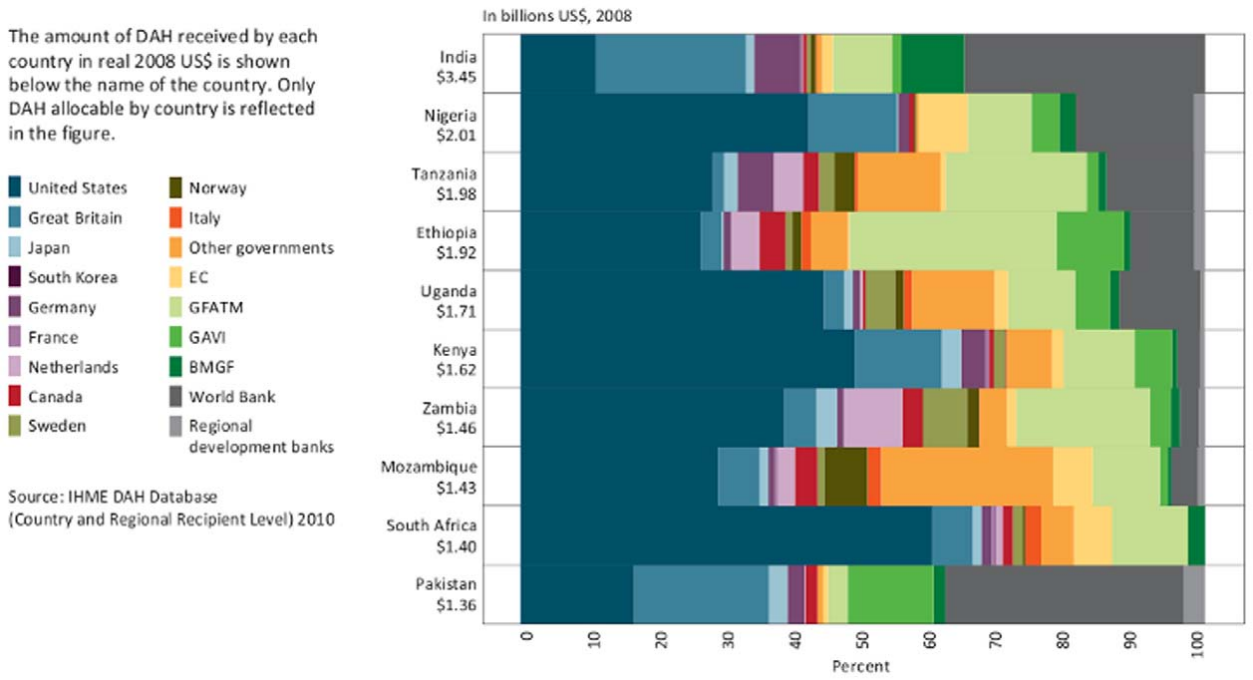
Top 10 recipients of DAH by percentage received from channels of assistance, 2003–2008.

However, the contribution of GF grants to total health expenditure (THE) within a given country varies considerably. In some countries, the GF makes a large contribution to overall health expenditure; while in others, it makes a small contribution. The relative contribution of the GF to all DAH also varies. Figure 1 describes the contributions of different donors amongst the ten largest recipients of DAH from 2003 to 2008 [2]. In India and Pakistan, the World Bank was the largest donor, accounting for about 35% of all DAH. But in Ethiopia, the Global Fund was the biggest single source of DAH, with the World Bank playing a relatively small role. And in Nigeria, Kenya and South Africa, the US and the UK governments together provided more than half of all DAH.

Historically, provided that applicants come from low and middle income countries, the Global Fund's approach to resource allocation has been based on a demand driven model. As a consequence, the Global Fund has had limited influence on the pattern of grant allocation. Over time however, the Global Fund has introduced policies to give it a greater influence over resource allocation whilst maintaining the demand-driven principle. These have included developing more refined eligibility criteria and expecting countries to demonstrate evidence of ‘counterpart financing’.

Presently, the Global Fund's eligibility criteria are as follows: low-income countries (LICs) and lower middle-income countries (MICs) are automatically eligible; while upper MICs have to demonstrate a ‘severe’ or ‘extreme’ generalised disease burden, or at least a ‘high’ concentrated burden of disease within a segment of the population. Additionally, grant proposals from lower MICs must focus at least 50% of the budget on underserved and most-at-risk populations and/or “highest impact interventions” while those from upper MICs must focus their entire budget on key populations and/or ‘highest impact interventions’.

The Global Fund's requirement for recipient countries to demonstrate ‘counterpart financing’ takes the form of: (a) a minimum threshold for government contributions to national disease programmes; (b) demonstration of increasing government contributions over time; and (c) improving the availability and reliability of health systems expenditure data. The minimum contribution is as follows: low income countries 5%; lower low MICs 20%; upper low MICs 40%; and upper MIC countries 60%.

Finally, the Global Fund employs a prioritisation framework in the event that there is insufficient money to cover all eligible *and* recommended funding proposals. This is based on a three-part composite index comprising income level, disease burden and TRP recommendation category in which greater priority is given to poorer and higher burden countries.

In recent months, the Global Fund has come under significant budgetary pressure. Its eleventh round of funding had to be cancelled. It is reviewing its approach to resource allocation with a view to reducing the number of countries eligible to apply for funding and tightening the criteria for the approval of grants. A High Level Panel that was established to investigate the Global Fund's fiduciary controls has also encouraged the GF to also incorporate an assessment of ‘financial risk’ in its approach to resource allocation [3].

We therefore undertook a study to examine how the GF's pattern of resource allocation related to: a) the level and pattern of total and government health expenditure; b) the income status of a country; c) the burden of disease related to HIV/AIDS, TB and malaria; and d) the potential to increase domestic public health financing by looking at the proportion of GDP raised as tax, and the proportions of the government budget spent on health and on the military. We then discuss how the Fund's resource allocation strategy can be improved.

## Methods

This is a cross-sectional study of all countries that received Global Fund disbursements in the year 2009. We collected data on GF disbursements in recipient countries between January 1^st^ and the end of December 2009 from spreadsheet files available on the GF website. We extracted data on the number of disbursements; the total amount of money disbursed; and whether the recipient was government or non-governmental. We used disbursement data rather than expenditure data because the former is more complete and accurate.

We only looked at single-country grants as it is not possible to determine the disbursements made to individual countries from multi-country grants. Eight multi-country grants with disbursements in 2009 were therefore excluded. A total of 110 countries and 2 ‘territories’ (Palestine and Zanzibar) received GF disbursements in 2009. However, because of incomplete and missing data, Kosovo, Somalia, Palestine, Zanzibar and Zimbabwe were excluded from further analysis; leaving 107 countries. Of these, three (Colombia, Costa Rica and Myanmar) received ‘*negative* disbursements’, meaning that monies were returned to the GF. In these countries, the GF did not therefore make a contribution to overall health financing and were consequently removed from further analysis; leaving 104 countries in the final results of this study.

Data on total health expenditure (THE), government health expenditure (GHE) and development assistance for health (DAH) for each country were obtained from the World Health Organisation's Global Health Observatory Data Repository (http://apps.who.int/ghodata) and its' Global Health Expenditure Database (http://apps.who.int/nha/database/PreDataExplorer.aspx?d=2). Data on military expenditure were obtained from the Stockholm International Peace Research Institute's Military Expenditure Database (http://www.sipri.org/databases/milex). Population data were obtained from the World Bank. Data on ‘general government final consumption expenditure as a percentage of GDP’ were obtained from the World Bank (http://data.worldbank.org/indicator/NE.CON.GOVT.ZS). This calculates all government current expenditures for purchases of goods and services (including compensation of employees) as a proportion of GDP, and is a rough measure of the proportion of GDP captured as revenue by the government. To measure the burden of disease, we adopted a methodology used by the Global Fund which designates a score of between 2 and 8 for each disease, which is then aggregated to form a composite score.

## Results

A total of 862 disbursements which amounted to US$ 2,604,733,440 were made to the 104 countries analysed. Of this amount, 63% (US$ 1,642,453,949) was disbursed to government recipients, and the remainder to non-government recipients. The average disbursement to government recipients (US$3.16 million) was slightly higher compared to the average disbursement to non-government recipients ($US2.81million). Only 39 of the 104 countries demonstrated dual-track financing in which disbursements are made to both government and non-government recipients.

In 37 countries (Albania, Azerbaijan, Bhutan, Botswana, Bulgaria, Cambodia, China, Congo, Djibouti, Egypt, Eritrea, Gabon, Georgia, Guinea, Guinea-Bissau, Guyana, Jamaica, Jordan, Kazakhstan, Kyrgyzstan, Laos, Lesotho, Macedonia, Malawi, Mongolia, Morocco, Mozambique, Namibia, Rwanda, Sierra Leone, South Africa, Swaziland, Timor-Leste, Tunisia, Uganda, Uzbekistan and Vietnam), all disbursements were made only to a government recipient; while in a further 8 countries (Benin, Cameroon, Central African Republic, Ethiopia, India, Moldova, Senegal and Suriname), more than 90% (but less than 100%) of the total amount of disbursements was made to a government recipient. At the other end of the spectrum, 28 countries (Belarus, Belize, Bolivia, Bosnia and Herzegovina, Brazil, Comoros, Congo (Democratic Republic), Cuba, Equatorial Guinea, Guatemala, Haiti, Honduras, Iran, Iraq, Liberia, Maldives, Mauritius, Montenegro, Nicaragua, Paraguay, Peru, Romania, Russian Federation, Sao Tome and Principe, Sudan, Syria, Tajikistan and Ukraine) had all their disbursements made to non-government recipients; while a further one country (Philippines) had more than 90% (but less than 100%) of the total disbursement amount made to non-government recipient(s).

**Figure 2:**
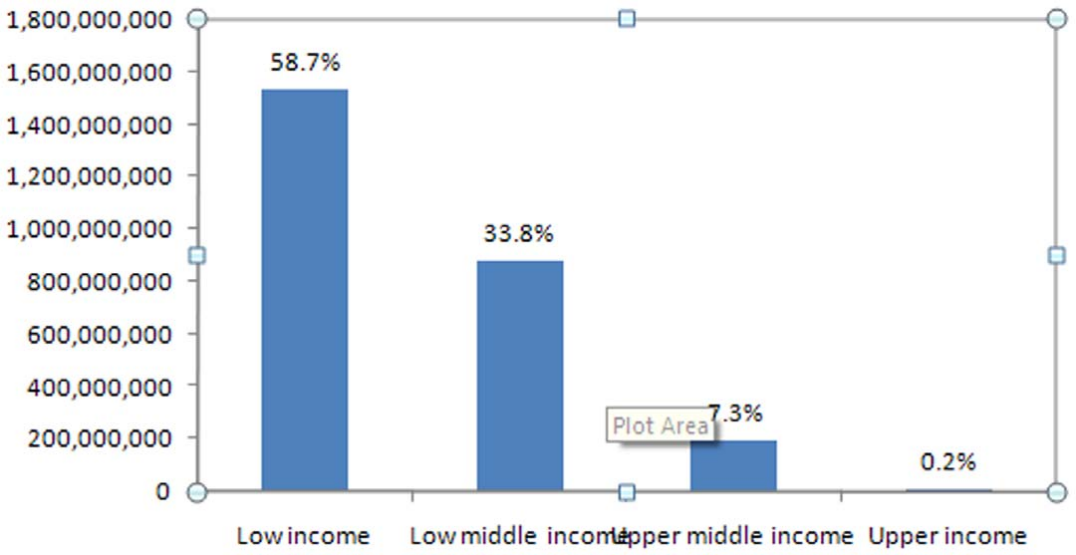
GF disbursements in 2009 by income group (US$).

The GF's disbursements by ‘income status’ in 2009 was progressive. Fifty-eight percent of its disbursements were to LICs; while a further 33.8% were to lower MICs (Figure 2).

Total GF disbursements in 2009 made up 0.37% of all health expenditure across the 104 countries. But their contribution to individual countries ranged from as little as 0.002% (Botswana) to 53.4% (Democratic Republic of Congo). Overall, the GF contributed 3.29% of THE in LICs, 0.22% in low MICs and 0.07% in high MICs. The fifteen countries with the highest GF contribution relative to THE are shown in Table 1 below. All these countries are classified as low income, except for Lesotho, Swaziland and Timor Leste which are low MICs.

**Table 1:** Fifteen countries with the highest Global Fund contribution relative to THE.

	Total GF disbursements as a % of THE
Congo (Democratic Republic)	53.4%
Gambia	29.3%
Eritrea	25.0%
Guinea-Bissau	23.2%
Malawi	23.0%
Burundi	16.9%
Rwanda	16.8%
Papua New Guinea	12.0%
Togo	11.3%
Ethiopia	10.7%
Lesotho	10.7%
Tanzania (United Republic)	9.6%
Swaziland	9.4%
Liberia	9.2%
Timor Leste	9.2%

Total GF disbursements in 2009 equalled about 0.74% of total *government* health expenditure (GHE) across the 104 countries; ranging from 0.003% in Botswana to 223% in the Democratic Republic of the Congo (where all the disbursements were made to non-government recipients). The fifteen countries with the highest GF contribution relative to total GHE are shown in Table 2 below. Five countries (Sierra Leone, Haiti, Laos, Cambodia and Tajikistan) from Table 2 do not appear in Table 1; while five countries from Table 1 are absent in Table 2 (Papua New Guinea, Lesotho, Tanzania, Swaziland and Timor Leste).

**Table 2:** Fifteen countries with the highest Global Fund contribution relative to total government health expenditure.

	Total GF disbursements as a % of total government expenditure on health
Congo (Democratic Republic)	223.0%
Guinea-Bissau	90.8%
Gambia	58.5%
Eritrea	56.1%
Togo	47.1%
Sierra Leone	41.2%
Malawi	39.6%
Rwanda	38.9%
Burundi	36.7%
Cambodia	34.1%
Haiti	32.9%
Laos	30.4%
Tajikistan	25.3%
Liberia	23.2%
Ethiopia	22.6%

As mentioned earlier, 63% of the value of all GF disbursements in 2009 was made to governments. GF disbursements to government amounted to 0.47% of GHE across the 104 countries, but with considerable inter-country variation. The fifteen countries with the highest GF contribution to government relative to total government health spending are shown in Table 3 below. In three countries, more than half the government's health expenditure appears to be based on Global Fund grants.

**Table 3:** Fifteen countries with the highest Global Fund contribution to government relative to total government health expenditure.

	GF disbursements to government as a percentage of total government expenditure on health
Guinea-Bissau	90.8%
Eritrea	56.1%
Gambia	52.0%
Sierra Leone	41.2%
Malawi	39.6%
Rwanda	38.9%
Cambodia	34.1%
Burundi	31.5%
Laos	30.4%
Ethiopia	21.8%
Uganda	17.8%
Lesotho	15.7%
Swaziland	14.9%
Togo	14.9%
Timor-Leste	12.9%

**Figure 3:**
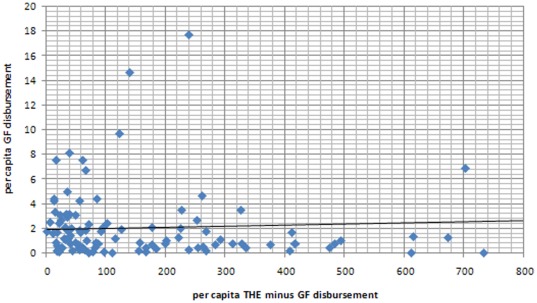
Scatter plot of per capita GF disbursements (US$ AER) and per capita THE minus per capita GF disbursement (US$ AER).

**Figure 4:**
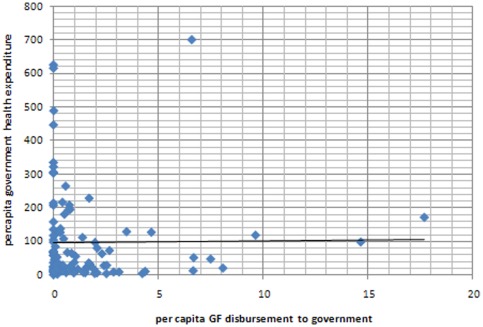
Scatter plot of per capita GF disbursements to government (US$ AER) and per capita government health expenditure (US$ AER).

Although we found a progressive pattern of funding across the different income groupings of countries, the absolute level of health funding from other sources does not appear to influence the GF's pattern of resource allocation. For example, we found no correlation between per capita GF disbursements and per capita THE minus Global Fund disbursements (Figure 3). Similarly, there was no apparent pattern in the relationship between per capita GF disbursement to government and per capita government health expenditure (Figure 4).

**Figure 5:**
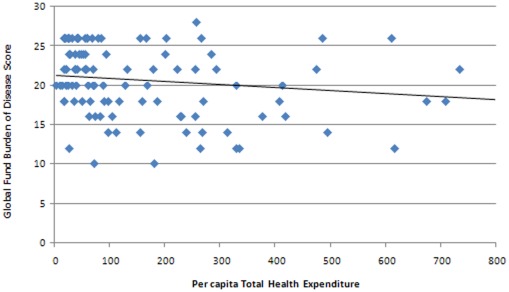
Scatter plot of per capita THE (US$ AER) and burden of disease score.

When we examined the relationship between burden of disease score with per capita THE we found that countries with a lower burden of disease score tended to have higher levels of THE (Figure 5). This is not surprising given the tendency for poorer countries to have a higher burden of disease.

**Figure 6:**
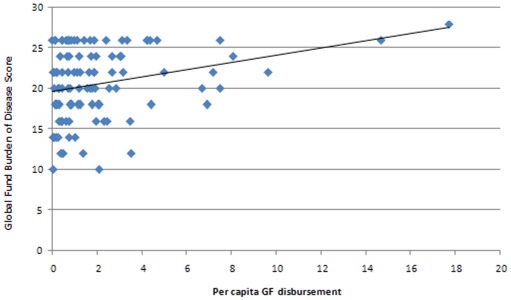
Scatter plot of per capita GF disbursement (US$ AER) and burden of disease score.

However, there was a positive correlation between per capita GF disbursement and the burden of disease score (Figure 6) which means that the GF does, to some degree, compensate for the low levels of spending in countries with a high burden of disease.

The twenty-two countries (and their governments) listed in Tables 1, 2 and 3 are all, to a lesser or greater extent, ‘dependent’ on the Global Fund in the sense that should the Global Fund stop making disbursements, there would be a significant financial impact on total and/or government health expenditure. Table 4 combines all the countries and data from Tables 1, 2 and 3 and adds six other data points: per capita THE; per capita GHE; THE as a percentage of GDP; GHE as a percentage of total government expenditure; DAH as a percentage of THE; and military expenditure as a percentage of government expenditure.

**Table 4:** Miscellaneous financing indicators of 22 selected recipients of Global Fund grants.

Country	Total GF disbursements as a % of THE	Total GF disbursements as a % of total government expenditure	GF disbursements to government as a % of total government expenditure on health	Per capita total health expenditure $US (AER)	Per capita government health expenditure $US (AER)	Total expenditure on health as a % of GDP	External resources for health as a % of THE	General government expenditure on health as a % of total government expenditure	Government Final Consumption Expenditure (AER $) as a % of GDP	Military expenditure as % government health expenditure
Burundi	16.9%	36.7%	31.5%	19.79	9.11	13.1	45.2	11.8		
Cambodia	7.3%	34.1%	34.1%	43.17	9.18	5.9	8.8	7.5	7.96	140.5%
Congo (DRC)	53.4%	223.0%	2.6%	3.33	0.80	2.0	36.0	1.7	7.90	231.6%
Eritrea	25.0%	56.1%	56.1%	10.12	4.51	2.2	65.6	3.1		
Ethiopia	10.7%	22.6%	21.8%	14.68	6.98	4.3	39.5	11.4	8.21	58.8%
Gambia	29.3%	58.5%	52.0%	25.63	12.84	6.0	26.3	11.6	15.94	
Guinea-Bissau	23.2%	90.8%	90.7%	18.36	4.69	6.1	42.0	4.0		
Haiti	7.3%	32.9%	85.0%	39.6	8.76	6.1	37.5	9.5		
Laos	5.8%	30.4%	30.4%	35.82	6.85	4.1	-	3.8		42.5%
Lesotho	10.7%	15.7%	29.7%	70.05	47.77	8.2	30.4	8.2		46.2%
Liberia	9.2%	23.2%	15.7%	29.36	11.64	13.2	47.0	17.2		15.6%
Malawi	23.0%	39.6%	39.6%	19.07	11.06	6.2	-	12.1	20.88	
Papua New Guinea	12.0%	17.4%	39.8%	41.59	28.83	3.54	17.6	8.0	10.69	20.3%
Rwanda	16.8%	38.9%	38.9%	48.18	20.83	9.0	53.2	16.8	14.65	36.2%
Sierra Leone	4.3%	41.2%	41.2%	45.5	4.78	6.2	19.7	6.4	13.76	
Swaziland	9.4%	14.9%	14.9%	155.78	98.65	6.3	12.2	9.3	27.02	86.4%
Tajikistan	8.4%	25.3%	14.8%	38	12.60	5.3	11.7	6.4	28.34	
Tanzania	9.6%	13.1%	0%	25.31	18.63	5.1	56.5	18.1	19.78	26.6%
Timor Leste	9.2%	12.9%	12.9%	73.24	51.99	12.3	-	9.8		64.5%
Togo	11.3%	47.1%	14.9%	27.29	6.52	5.5	18.5	6.4		
Uganda	3.4%	17.8%	17.8%	42.55	8.06	8.2	20.9	11.6	11.39	120.4%

Amongst these countries, per capita THE varies from US$3.33 in the DRC to US$155.78 in Swaziland. Thus, although these countries may all be relatively ‘dependent’ on the Global Fund, those with low levels of per capita THE will be more reliant on Global Fund grants in absolute terms for the provision of basic and essential of health care. Table 4 also shows considerable differences in the contribution of all DAH to THE, ranging from 8.8% in Cambodia to 65.6% in Eritrea. There was also a ten-fold difference in GHE as a percentage of total government expenditure between the DRC (1.7%) and Tanzania (18.1%).

Only 12 countries in Table 4 had data on ‘government final consumption expenditure as a percentage of GDP’, with figures ranging from 7.9% to 28.3%, with an average of 15.5%. This is surprisingly high compared to the average across all low income countries which is 10.0%. Only 12 countries in Table 4 had data on military expenditure of which three (Cambodia, DRC and Uganda) spent more on the military than on health.

These data however reveal most information when examined on a country by country basis. For example, per capita THE in the DRC was only US$3.33 in 2009, of which only US$0.80 came from government budgets. By contrast, the Global Fund contributed more than half (53.4%) of all health expenditure, and nearly double the amount spent by the government. However, THE as a percentage of GDP was only 2% and Government Final Consumption Expenditure as a % of GDP was 7.9%, which suggests that there is the potential to expand the domestic funding base for health. Furthermore, government expenditure on health as a % of total government expenditure was only 1.7% (far from the Abuja Declaration target of 15%) and military expenditure was more than double that of government health expenditure, both of which indicate further potential to increase domestic public spending on health.

The case of the Gambia offers a contrast. Here, the Global Fund contributes 29.3% of all health expenditure. However, THE as a percentage of GDP is 6% (three times that of the DRC) while Government Final Consumption Expenditure as a % of GDP is 15.9% (more than double that of the DRC). And government health expenditure as a % of total government expenditure is 11.6%, which is nearly seven times more than the DRC and not far from the Abuja Declaration target of 15%. The potential to expand the domestic funding base for health in Gambia is therefore less than in the DRC.

Finally, in the case of Liberia, 9.2% of THE was funded by the Global Fund. This is a much smaller proportion than Gambia even though per capita THE and the burden of disease score in Liberia is similar to that of Gambia. However, external sources of funding made up 47% of THE in Liberia which means that other donors played a bigger role in supporting the Liberian health system than in the Gambia. Total health expenditure as a proportion of GDP is relatively high (13.2%) as is GHE as a proportion of total government expenditure (17.2%) which suggests less potential for increasing domestic finance for health in Liberia compared to the Gambia and DRC.

## Discussion

The findings described above raise a number of issues about the Global Fund's contribution to health spending in low and middle income countries. Across the 104 countries that were studied, total GF disbursements in 2009 made up only 0.34% of THE, while GF disbursements to government made up 0.47% of GHE. Overall, the Fund's contribution to health improvement is therefore relatively small.

However, on average, the GF's contribution to poorer countries is higher. Global Fund disbursements made up 3.29% of THE and 5.4% of GHE across all LICs. In several countries, GF disbursements accounted for more than 10% of THE; and in the DRC, the sum of GF disbursements was more than twice the amount spent by the government on health. In Guinea-Bissau, Eritrea and the Gambia, Global Fund disbursements to government contributed to more than 50% of total GHE. This country-by-country variation in the degree to which the Global Fund supports health financing mirrors a pattern that has been observed for overall DAH. For example, in 2006 DAH was estimated to have contributed an average of $6 per capita to all low-income countries (amounting to about just under 25% of THE); but the variation in per capita DAH ranged from $0.50 to $27.77 [4].

Although, the Global Fund's allocation of funds is progressive when examined across the three main income groups (LICs, lower MICs and upper MICs), we found a surprising lack of correlation between the Global Fund's financial support and the level of health financing at an individual country level. No relationship was found between per capita GF disbursements and per capita THE (Figure 3), nor between per capita Global Fund disbursements to government and per capita GHE (Figure 4). In other words, levels of health spending from other sources within a country do not appear to influence the Global Fund's pattern of resource allocation.

Since 2009, a number of LICs have become classified as lower MICs and a number of lower MICs have become upper MICs. Thus the amounts spent in MICs in 2010 and 2011 will almost certainly have increased relative to LICs. This may not be inappropriate as there is now a poor correlation between the global distribution of people living in poverty and suffering from the three diseases, with the official income status of countries. For example, more than 70% of the world's poor (living on less than an income of $1.25 per day) now live in MICs (the majority in Pakistan, India, Nigeria, China and Indonesia). MICs also now have a larger total disease burden than LICs [5].

The extent to which the Fund's rules for counterpart financing influence the pattern of resource allocation could not be examined because of the lack of data on government spending on disease programmes, as well as the lack of a clear and agreed approach for allocating ‘cross-cutting’ government expenditure on health (e.g. on logistical and management systems or the salaries of generic health workers) to specific disease programmes. This raises questions about the validity and feasibility of making a distinction between disease-based budgets and expenditure, and other budget and expenditure categories that relate to the overall functioning of the health system but which make an indirect contribution to disease-based programmes.

In our study, 28 countries (27%) had all disbursements made to a non-government recipient; while considerably more countries had PRs that were entirely or predominantly governmental. We found no obvious difference between these two sets of countries, although an earlier study found that Global Fund grants to ‘fragile states’ were mainly channelled to non-governmental entities [6]. The country-led funding model of the Global Fund means that the choice of putting forward a government or non-government PR will be mostly due to country-level factors rather than to any strategic choice made by the Global Fund.

In spite of the demand-led nature of the funding model, a positive correlation was found between per capita Global Fund disbursements and burden of disease. This will have been partly due to the Fund's eligibility criteria. However, the correlation not strong, and echoes findings from another analysis which concluded that the Global Fund needed to improve the alignment of its grants for malaria control with the epidemiological pattern of malaria [7].

Currently, the funding model which determines the Global Fund's pattern of resource allocation consists of four components. The first are the demand-driven or country-led factors which determine if a country decides to apply to the Global Fund or not, and what goes into the grant applications. The second are quality and performance-related factors comprising the quality of the grant application which has to gain the approval of the TRP, and subsequent grant performance which influences the rate and completeness of grant disbursements. The third are the Global Fund's eligibility and prioritisation criteria (based on a country's income status and its disease burden) which provides an element of supply-led resource allocation that is needs based. And the fourth component is the Global Fund's counterpart financing policy which is designed to leverage government spending on the three diseases.

Thus, the pattern of resource allocation is the outcome of a mix of various demand-driven and supply-led factors, each of which the Global Fund itself has variable degrees of influence. A key question is whether the funding model can or should be improved in any way.

For example, one recommendation might be to incorporate a measure of ‘financial risk’ as an additional component of the funding model to reduce the Fund's exposure to fraud or corruption by limiting the amount of funding to countries considered to be high risk. In our view, this would be inappropriate. While an assessment of ‘financial risk’ should be conducted to influence the design of financial procedures and fiduciary controls, the Fund's resource allocation strategy should be based primarily on a combined assessment of health and financial need. Furthermore, those countries with weak financial management systems and inadequate fiduciary controls are often those in need of additional DAH and long-term systemic developments.

But if the Global Fund is to improve the alignment of its grants to a country's ‘need’, it would need to reduce the relative importance of demand-driven and country-led factors. As the resource envelope shrinks and as demand increasingly outstrips the supply of money, the current model will become more competitive. Grant applicants competing over a more constrained budget will force the Global Fund to apply more stringent rationing. But the Fund could go in one of two directions. It could lean towards the quality and performance-related factors, and decide to preferentially allocate funds to better applications and better track records of past performance; or it could lean towards prioritising grant applications on the basis of need.

In terms of the latter, the Global Fund's approach has been to use a country's income status to help it prioritise poor countries, and to use a measure of burden of disease to help it allocate resources to countries with lots of disease. However, a more sophisticated approach may now be required. In the case of assessing a country's burden of disease, this is straightforward provided there are good data. And where this is lacking, the Global Fund is already working with other development partners to improve disease surveillance. But because aggregated measures of disease burden at the country level can mask the particular needs of minority and marginalised population groups, the Global Fund will need to ensure that such groups continue to receive appropriate external financial support, not so much because of the country's level of financial need, but for socio-political reasons.

When it comes to assessing the financial need of a country, the current funding model may need more substantial modification because the income status of a country is a poor indicator of a country's need for financial support. This is due in part to the discrepancy between income status and the prevalence of household poverty, and to the fact that the cost of an adequate and universal response to HIV/AIDS, TB and malaria programmes, especially in high burden countries, can be challenging, if not unaffordable, even for middle income countries.

Ideally, a detailed assessment of the actual *cost* of providing treatment and prevention services in specific countries would form the basis of assessing a country's need for external assistance (taking account of factors such as the degree and scale of poverty amongst diseased or at-risk population groups; the unmet need associated with other diseases, illnesses and health threats; population density; the availability and quality of existing health care infrastructure; and the price of key health systems inputs and technologies).

Furthermore, a country's need for external financial assistance is not fixed. As shown in this study, many countries have the *potential* to expand domestic financing for HIV/AIDS, TB and malaria programmes. Assessing and measuring the size of this potential, as well as the feasibility of realising this potential, requires an examination of: the adequacy of THE and government health budgets; the contribution of other donors; the potential for increasing government revenue streams (e.g. through more efficient and effective tax systems); and the potential to increase the allocation of government budgets to health (e.g. by diverting spending away from armaments). In addition, social and political factors inhibiting an adequate or appropriate domestic response to the three diseases would need to be considered.

Finally, in many countries, out-of-pocket expenditure makes up a high proportion of THE (nearly half in some cases). This is a form of financing does not permit cross-subsidisation or risk pooling, and which is inefficient. Thus, the potential for a country to make efficiency savings and equity gains through schemes to pool health finance should also be assessed.

This is where the policy on counterpart financing becomes relevant. Counterpart financing was primarily designed to avoid or minimise a certain form of moral hazard in which funding from the Global Fund would cause governments to abscond or weaken their own obligations to fund HIV, TB and Malaria programmes. However, it is also a strategy that could encourage positive health systems improvements that extend beyond merely encouraging governments to co-finance HIV, TB and Malaria programmes.

Such improvements might include expanding the domestic funding base for health care and getting governments to raise their budgetary allocations for health more generally. While not having a direct impact on improving the coverage of HIV, TB and Malaria programmes, they have the potential to make positive indirect impacts on such programmes *and* to improve their prospects for future financial sustainability.

However, the Global Fund's current counterpart policy is narrowly focused on the three diseases, and is also not without some potential drawbacks. For example, it might inadvertently result in the preferential allocation of funds to countries that are already spending money on HIV/AIDS, TB and Malaria, rather than getting the GF to compensate for low or poor levels of domestic funding. Here lies a tension for the Global Fund. On the one hand, it wants to use its grant-making power to leverage more government health spending on HIV/AIDS, TB and Malaria. On the other hand, its mission to respond to *people* in need may require the Fund to approve grants in spite of or because of government neglect.

The counterpart financing policy may also threaten to distort local priority setting. For example, through the Global Fund (and other donors), the three diseases of HIV, TB and Malaria may already be well-funded or even over-funded relative to other priorities. The Fund's counterpart financing policy might inadvertently accentuate this problem. Thus, the current counterpart financing rules are on the one hand too generalised in that they fail to accommodate the diverse and heterogeneous nature of countries and governments; whilst on the other hand, are too narrow and specific, in that they are focused only on government funding for three diseases.

### Conclusions and recommendations

The Global Fund is merely ten years old. In its first decade of existence it developed a funding model that was designed to be quick, pragmatic and country-driven. Its' mission was to get money out quickly and rapidly translated into the uptake of services and treatments for HIV/AIDS, TB and Malaria. The Fund is now entering a phase that will require it to allocate its finite funds more carefully and strategically. Although its funding model already incorporates some explicit needs-based resource allocation, this paper suggests that some modifications are in order.

Given current financial realities, the Fund must first address the tension between being a responsive, demand-driven funder and being a more directive, supply-led funder. The Global Fund's strategy for 2012–16 and its Consolidated Transformation Plan indicate that it is moving towards becoming a more supply-led funder. They also indicate that the Fund will develop a more iterative, hands-on and country-specific process for providing future financial support to countries. In theory, this makes sense from a technical and public health perspective, but it poses potential threats to the benefits that are derived from the demand driven and country-owned process of countries applying for grants through periodic funding rounds.

Managing this tension between being responsive to country-led applications and responding to the health needs of populations and the financial needs of countries may require a more sophisticated multi-track approach. One track might entail the Global Fund providing funds to countries that need assistance and capacity development in producing sound and appropriate HIV, TB and Malaria plans. A second track would entail the continuation of the country-led model of having countries apply for grants. And a third track might entail setting of country-specific budget ceilings and floors based on the Fund's own budget, as well as an assessment of the combined financial and health needs of recipient countries. But as suggested earlier, this third track should involve a more sophisticated assessment of health and financial need than is currently the case.

As far as counterpart financing is concerned, the Global Fund should revise its policy so that it uses its grant-making powers to leverage improvements in health systems financing overall, rather than to just increase domestic financing for three diseases. At the same time, it would need to ensure that the counterpart financing requirement does not inadvertently penalise communities in need of support because their governments have failed to make adequate commitments to health. This too would be enabled by the Global Fund adopting a more supply-led funding model in which it could be more directive in channelling funds to non-governmental recipients.

In addition to raising questions about the tension between being a demand-driven and supply-led donor, this discussion also implicitly refers to the tension between being a disease-based donor and needing to take a more comprehensive health systems perspective. As funding becomes constrained and questions about financial sustainability and efficiency become more important, the Fund must consider how it catalyse the *systemic developments* that are necessary for securing the long term sustainability, effectiveness and efficiency of HIV/AIDS, TB and Malaria programmes. This should include revisiting and improving the Fund's approach towards funding health systems strengthening and community systems strengthening activities.

But because the Global Fund has a narrow remit and because it is one of several donors and actors that impact upon the health systems of low and middle income countries, such an approach towards strategic funding and systems strengthening, can only be done effectively if the Global Fund is either able to work more effectively in concert with other development partners, or expand its scope and remit. What is clear however, from both the research findings and discussion of this paper is that it is simply inappropriate to examine the Global Fund's financing and impact in isolation of other key determinants of health systems performance.
